# Effect of yeast cell wall supplementation on production performances and blood biochemical indices of dairy cows in different lactation periods

**DOI:** 10.14202/vetworld.2019.796-801

**Published:** 2019-06-13

**Authors:** Min Aung, Hiromichi Ohtsuka, Kenichi Izumi

**Affiliations:** 1 Department of Animal Nutrition, University of Veterinary Science, Nay Pyi Taw 15013, Myanmar; 2 Department of Veterinary Medicine, School of Veterinary Medicine, Rakuno Gakuen University, Ebetsu 069-8501, Japan; 3 Department of Sustainable Agriculture, College of Agriculture, Food and Environment Sciences, Rakuno Gakuen University, Ebetsu 069-8501, Japan

**Keywords:** dairy cows, electrolyte indices, energy metabolites, liver enzyme activity, milk yield, yeast cell wall

## Abstract

**Aim:**

This experiment was conducted to determine the effect of yeast cell wall (YCW) supplementation on production performances and blood biochemical indices such as liver enzyme activities, energy metabolites, and electrolyte concentrations of dairy cows in different lactation periods (LP).

**Materials and Methods:**

Thirty-two lactating Holstein cows were assigned into 2×2 factorial arrangement, in which the factors were the treatment (TM) (control [n=16] vs. YCW [n=16]) and the LP (early lactation [n=14] vs. mid-lactation [n=18]). The cows with day in milk (DIM) <120 (81±7 DIM) were defined as early lactating cows, whereas the cows with DIM >120 (179±5 DIM) were assumed as mid-lactating cows. The YCW (SafMannan; Phileo, Lesaffre Animal Care, France) was used as the dietary supplement (10 g/cow/day) in this experiment. The statistical analysis of the data was performed by the two-way analysis of variance using the general linear model procedure to determine the main effects (TM and LP) and their interaction (TM×LP) on production performances and blood biochemical parameters of experimental cows.

**Results:**

No significant effects (p>0.05) of YCW supplementation on production performances and blood biochemical indices of cows in TM groups (control vs. YCW) were observed; however, some obvious effects were detected in LP (early- and mid-lactation). Milk and milk component yield of cows in early lactation were significantly higher (p<0.05) than in mid-lactation, whereas somatic cell count and milk urea nitrogen were not different (p>0.05) with the YCW supplementation. The higher level (p<0.05) of serum albumin was found in mid-lactating cows after YCW supplementation. Before the experiment, the higher (p<0.05) non-esterified fatty acid (NEFA) and NEFA/total cholesterol (T-Cho) ratio, and the lower (p<0.05) calcium (Ca) concentration were observed in early lactating cows comparison with mid-lactating cows; however, there were not different after YCW supplementation.

**Conclusion:**

The positive effects of YCW supplementation on milk and milk component yields, energy metabolite, especially NEFA and NEFA/T-Cho ratio and Ca concentration were observed in early lactating cows rather than mid-lactating cows.

## Introduction

Early lactation in cows is both physiologically stressful and a period of high milk yield, during which cows may experience both low feed intake and lower immune function [1]. This can lead to net negative energy intake and low production performance [2]. Yeast supplementation, particularly in the form of the yeast cell wall (YCW), can provide effective immunomodulation, with antioxidant, and anti-inflammatory activities [3] and may reduce the impact of stress factors and enhance immune status during early lactation. Yeast products have the ability to enhance the population and efficiency of ruminal microbes, resulting in increased fiber digestion and altered volatile fatty acid production [4], thereby improving ruminal fibrobacterial activity and increasing feeding efficiency. YCW contains polysaccharides such as mannan and β-glucan. Mannan oligosaccharide (MOS) is capable of binding to receptors of pathogenic bacteria, preventing adhesion, and colonization of the gastrointestinal tract of lactating dairy cows [5]. The β-glucans (β-1,3 and β-1,6-glucan) can stimulate the release of cytokines from macrophages in mice [6]. Thus, YCW supplementation contributes to overall digestive tract health and improves immune system function, which, in turn, contributes to improved animal performance.

The dietary application of yeast culture and YCW as feed supplement can improve ruminal microbial activity in the Rusitec system [7], and ruminal fiber digestion in cattle [4]. Feed intake and milk yield are increased with the supplementation of yeast culture in transition cows [8]. In experiments on early lactating dairy cows, the supplementation of MOS alone or in combination with live yeast and therapeutic use of β-glucans infused into infected quarters had no effect on production performance [9] and chronic subclinical mastitis [10], respectively. In contrast, the dietary supplementation of yeast culture and enzymatically hydrolyzed yeast increased milk production, milk quality, and mammary gland health in early lactating cows [5]. The dissimilarity between these reports indicates that the effects of yeast products’ supplementation on production performances and udder health of early lactating dairy cows still require investigation. Reports concerning the effect of YCW supplementation on production performance and biochemical indices of dairy cows during different lactation periods (LPs) are also limited.

This experiment was conducted to determine the effect of YCW supplementation on production performances and blood biochemical indices such as liver enzyme activities, energy metabolites, and electrolyte concentrations of dairy cows in different LPs.

## Materials and Methods

### Ethical approval

This work was approved by the Animal Experiment Committee of Rakuno Gakuen University for the use of animals in experiments.

### Experimental animals and design

Thirty-two lactating Holstein cows were assigned into 2×2 factorial arrangement, in which the factors were the treatment (TM) (control [n=16] vs. YCW [n=16]) and the LP (early lactation [n=14] vs. mid-lactation [n=18]). The cows with day in milk (DIM) <120 (81±7 DIM) were defined as early lactating cows, whereas the cows with DIM >120 (179±5 DIM) were assumed as mid-lactating cows. Thus, each TM group consists of a total of 16 cows from two different LPs (early lactation [n=7] and mid-lactation [n=9]). For the LP groups, early lactation group consists of 14 cows (control [n=7] and YCW [n=7]) and mid-lactation group consists of 18 cows (control [n=9] and YCW [n=9]). The experimental cows for each group were adjusted according to parity, DIM, milk yield, milk fat, and milk protein contents.

### Feeds and management

Total mixed ration (TMR) with the inclusion of 27% corn silage, 28% grass silage, 6% beet-pulp, 9% stream rolled corn, 11% soybean meal, 6% soy-sauce meal, 6% concentrate mixture, 5% wheat flour, 0.9% mineral supplement, and 0.1% vitamin supplement as dry matter basis was used as a basal diet. Its chemical compositions were 14.33% crude protein (CP), 37.33% neutral detergent fiber, and 70.83% total digestible nutrient (TDN). The powder form of the cell wall of yeast (*Saccharomyces cerevisiae*) (SafMannan; Phileo, Lesaffre Animal Care, France) was used as the dietary supplement in this experiment. The cows from the control group were fed TMR only and the cows from the YCW group were fed TMR with 10 g/cow/day YCW supplementation.

Cows were fed TMR *ad libitum* to allow for 5-10% refusal. Refused feeds were removed at 09:00 h and TMR was delivered at 10:00 h for the entire experimental period. The experiment lasted 10 weeks, with the first 2 weeks regarded as the adaptation period and the last 8 weeks regarded as the experimental period.

Cows were housed in a free-stall barn covered with rubber chip mattresses, on which wheat straw was spread as bedding. A mineralized salt block and water were freely available at all times. This experiment was carried out at the Rakuno Gakuen Field Education and Research Center (Rakuno Gakuen University, Ebetsu, Hokkaido, Japan).

### Measurements and sample collection

Feed intake, milk production and composition, and blood biochemical parameters were measured regularly throughout the experimental periods. Samples of feeds and refusals were collected and weighed for 2 consecutive days every week to determine the herd’s dry matter intake (DMI). The individual DMI for a cow was estimated by dividing the herd’s DMI by the number of cows in each group. Cows were milked at 05:30 and 16:00 h every day. Milk yields were automatically recorded at each milking and samples were taken for 2 consecutive days (in the evening of the 1^st^ day and morning of the 2^nd^ day) every week for composition analysis. Fat-corrected milk and energy-corrected milk were calculated according to the following equations: 4% Fat corrected milk (FCM)=0.4×(milk yield)+15×milk fat [11] and energy corrected milk (ECM)=(12.82×milk fat)+(7.13×milk protein)+(0.32×milk yield) [12], with all units as measured in kg yield. Before morning feeding, blood samples were obtained from the coccygeal vein and placed in evacuated serum-separating tubes and stored in the refrigerator until analysis. Samples were collected 2 times, before the experiment (week 0) and after the experiment (week 8), for biochemical analysis.

### Laboratory analysis

Feeds and refusals were dried at 60°C in a forced air oven for 48 h to estimate the DMI of experimental cows. Chemical composition of forage and TMR was analyzed by the Agricultural Product Chemical Research Laboratory in the Tokachi Federation of Agricultural Cooperatives using near-infrared spectrophotometry. The concentration of total digestible nutrients (TDN) in forage and TMR was calculated using estimated equations of NRC [13]. Milk samples were analyzed individually by the Hokkaido Dairy Milk Recording and Testing Association using near-infrared spectrophotometry. Blood biochemistry was measured at a commercial laboratory (Daiichi Kishimoto Clinical Laboratory Inc.). Albumin (ALB), gamma-glutamyl transferase (GGT), magnesium (Mg), calcium (Ca), and inorganic phosphorus (IP) were analyzed using colorimetry. To analyze blood urinary nitrogen (BUN), urease method was utilized. Blood glucose was analyzed using a hexokinase method. Total ketone bodies (TK) were analyzed using an enzyme cycling method. To determine non-esterified fatty acid (NEFA) and total cholesterol (T-Chol), an enzymatic assay was used. Aspartate aminotransferase (AST) was analyzed using a malate dehydrogenase-Ultraviolet method. Sodium (Na), chlorine (Cl), and potassium (K) were analyzed using an ion selective electrode method.

### Statistical analysis

The statistical analyses of the data were performed by the two-way analysis of variance (two-way ANOVA) using the General Linear Model procedure to determine the main effects, TMs (control vs. YCW) and LPs (early lactation vs. mid-lactation), and their interaction (TM×LP) on production performances and blood biochemical parameters of experimental cows. The Statistical Package for the Social Sciences (SPSS) [14] for Windows version 16.0 (Chicago, SPSS Inc.) was used for all statistical procedures. Differences were considered significant at p<0.05.

## Results

The significant interactions (p>0.05) between TM and LP of the production performances, liver enzyme activity, energy metabolites, and blood electrolyte indices of dairy cows were not observed (Tables-1-4).

**Table-1:** Effect of YCW supplementation on production performances of dairy cows in different LPs.

Variables	Treatments (Mean±SE)				p-value		

	Control		YCW				
	
	Early lactation	Mid lactation	Early lactation	Mid lactation	TM^†^	LP^††^	TM*LP
DMI (kg/d)	21.59		21.64		-	-	-
Milk yield							
kg/d	25.52±1.96	24.48±1.73	28.97±1.54	22.83±1.82	0.627	0.028	0.117
4% FCM	25.18±1.83	24.66±1.62	28.95±1.44	22.92±1.70	0.585	0.026	0.063
ECM	27.01±1.96	26.80±1.73	30.98±1.54	24.86±1.82	0.603	0.040	0.065
Milk composition (%)							
Fat	3.93±0.17	4.11±0.15	4.02±0.14	3.99±0.16	0.856	0.623	0.451
Protein	3.31±0.09	3.58±0.08	3.30±0.07	3.48±0.09	0.611	0.016	0.693
Lactose	5.20±0.09	5.19±0.08	5.27±0.07	5.04±0.08	0.715	0.263	0.318
SNF	8.74±0.14	9.00±0.12	8.83±0.11	8.77±0.13	0.650	0.308	0.347
Milk component yield (kg/d)							
Fat	1.00±0.08	0.99±0.07	1.16±0.06	0.92±0.07	0.576	0.037	0.054
Protein	0.84±0.06	0.87±0.05	0.95±0.05	0.80±0.06	0.674	0.160	0.083
Lactose	1.33±0.11	1.27±0.09	1.48±0.08	1.16±0.10	0.842	0.035	0.136
SNF	2.22±0.17	2.20±0.15	2.55±0.14	2.02±0.16	0.633	0.047	0.087
Somatic cell count (SCC)							
Count (10^4^/ml)	20.56±10.03	26.54±8.84	10.87±7.86	11.38±9.30	0.130	0.548	0.684
Index (log_10_)	1.09±0.20	1.11±0.18	0.87±0.16	1.14±0.19	0.570	0.395	0.522
MUN (mg/dL)	11.98±0.41	12.66±0.37	12.28±0.32	12.77±0.38	0.556	0.431	0.557

DMI=Dry matter intake, FCM=Fat corrected milk, ECM=Energy corrected milk, SNF=Solid non-fat, SCC=Somatic cell count, MUN=Milk urea nitrogen, YCW=Yeast cell wall, SE=Standard error,

† p value for treatment (TM) groups (control vs. YCW),

†† p value for lactation period (LP) (early lactation vs. mid lactation)

**Table-2:** Effect of yeast cell wall supplementation on liver enzyme activity of dairy cows in different LPs.

Variables	Sampling time	Treatments (Mean±SE)				p-value		

		Control		YCW				
	
		Early lactation	Mid lactation	Early lactation	Mid lactation	TM^†^	LP^††^	TM*LP
ALB (g/dL)	Before	2.92±0.07	3.08±0.05	3.18±0.07	3.09±0.05	0.082	0.571	0.057
	After	3.04±0.04	3.18±0.03	3.13±0.04	3.21±0.03	0.129	0.004	0.449
AST (U/L)	Before	93.60±6.52	85.67±4.86	87.75±7.29	83.50±5.16	0.514	0.324	0.763
	After	76.10±4.40	72.06±3.28	78.75±4.92	80.06±3.48	0.205	0.741	0.518
GGT (U/L)	Before	37.20±7.79	29.11±5.80	26.50±8.70	28.38±6.16	0.436	0.671	0.497
	After	27.20±3.98	28.00±2.96	27.38±4.45	27.38±3.14	0.952	0.915	0.915
BUN (mg/dL)	Before	10.56±0.86	11.20±0.64	11.05±0.96	11.13±0.68	0.797	0.658	0.726
	After	11.52±0.88	12.37±0.66	10.96±0.99	11.01±0.70	0.252	0.589	0.626

Alb=Albumin, AST=Aspartate aminotransferase, GGT=Gamma-glutamyl transferase, BUN=Blood urea nitrogen, YCW=Yeast cell wall, SE=Standard error,

† p value for treatment (TM) groups (control vs. YCW),

†† p value for lactation period (LP) (early lactation vs. mid lactation)

**Table-3:** Effect of yeast cell wall supplementation on energy metabolites of dairy cows in different LPs.

Variables	Sampling time	Treatments (Mean±SE)				p-value		

		Control		YCW				
	
		Early lactation	Mid lactation	Early lactation	Mid lactation	TM^†^	LP^††^	TM*LP
BG (mg/dL)	Before	64.00±1.47	61.56±1.10	61.25±1.65	63.57±1.16	0.840	0.984	0.083
	After	64.90±1.02	63.39±0.76	65.38±1.13	64.81±0.80	0.323	0.282	0.619
T-Cho (mg/dL)	Before	166.20±14.05	189.11±10.47	137.00±15.70	161.88±11.11	0.041	0.080	0.941
	After	212.20±18.62	212.83±13.88	165.25±20.82	166.12±14.72	0.013	0.966	0.994
NEFA (mg/dL)	Before	0.134±0.02	0.092±0.01	0.133±0.02	0.095±0.02	0.971	0.032	0.903
	After	0.083±0.004	0.082±0.003	0.079±0.004	0.080±0.003	0.425	0.991	0.726
NEFA/T-Cho	Before	0.031±0.004	0.019±0.003	0.036±0.005	0.024±0.003	0.228	0.006	0.886
	After	0.016±0.002	0.016±0.002	0.019±0.002	0.020±0.002	0.080	0.848	0.752
TK (mmol/L)	Before	502.60±53.68	492.22±40.01	483.50±60.02	518.88±42.44	0.940	0.804	0.650
	After	605.60±59.34	691.00±44.23	624.88±66.34	645.88±46.91	0.816	0.344	0.564
ACAC (mmol/L)	Before	8.60±3.63	9.67±2.71	9.25±4.06	6.88±2.87	0.753	0.848	0.614
	After	15.90±3.33	14.28±2.48	16.25±3.73	12.31±2.64	0.796	0.378	0.711
BHB (mmol/L)	Before	494.00±51.05	482.56±38.05	474.25±57.08	512.00±40.36	0.919	0.783	0.608
	After	589.70±56.57	676.72±42.17	608.62±63.25	633.56±44.73	0.819	0.297	0.560

BG=Blood glucose, T-Chol=Total cholesterol, NEFA=Non-esterified fatty acid, TK=Total ketone, ACAC=Acetoacetate, BHB=Beta-hydroxybutyrate, YCW=Yeast cell wall, SE=Standard error,

† p value for treatment (TM) groups (control vs. YCW),

†† p value for lactation period (LP) (early lactation vs. mid lactation)

**Table-4:** Effect of yeast cell wall supplementation on blood electrolyte indices of dairy cows in different LPs.

Variables	Sampling time	Treatments (Mean±SE)				p-value		

		Control		YCW				
	
		Early lactation	Mid lactation	Early lactation	Mid lactation	TM^†^	LP^††^	TM*LP
Na (mEq/L)	Before	138.40±0.67	138.44±0.50	138.00±0.75	138.50±0.53	0.784	0.665	0.717
	After	138.70±0.69	139.28±0.52	138.62±0.77	138.69±0.55	0.608	0.622	0.691
K (mEq/L)	Before	4.60±0.12	4.90±0.09	4.60±0.13	4.65±0.09	0.266	0.125	0.266
	After	4.78±0.13	4.70±0.10	4.71±0.14	4.73±0.10	0.879	0.797	0.678
Cl (mEq/L)	Before	96.40±0.86	98.00±0.64	97.00±0.96	97.38±0.68	0.988	0.227	0.449
	After	97.40±0.68	98.39±0.51	97.25±0.76	98.44±0.54	0.936	0.097	0.876
Ca (mg/dL)	Before	9.64±0.15	9.96±0.20	9.53±0.16	10.25±0.22	0.646	0.009	0.276
	After	9.79±0.15	9.69±0.11	9.71±0.17	9.66±0.12	0.698	0.580	0.874
P (mg/dL)	Before	5.38±0.27	5.12±0.20	5.38±0.30	5.36±0.21	0.644	0.596	0.630
	After	6.01±0.19	5.74±0.14	5.60±0.21	5.47±0.15	0.066	0.274	0.708
Mg (mg/dL)	Before	2.16±0.10	2.27±0.08	2.23±0.11	2.21±0.08	0.955	0.624	0.535
	After	2.36±0.06	2.34±0.05	2.35±0.07	2.47±0.05	0.325	0.374	0.250

Na=Sodium, K=Potassium, Cl=Chloride, Ca=Calcium, P=Phosphorus, Mg=Magnesium, YCW=Yeast cell wall, SE=Standard error,

† p value for treatment (TM) groups (control vs. YCW),

†† p value for lactation period (LP) (early lactation vs. mid lactation)

The summarized effects of YCW supplementation on production performances of dairy cows in different LPs are presented in Table-1. No remarkable change in DMI (kg/d) was observed in cows from either group. The production performances (milk yield, milk composition and component yield, somatic cell count [SCC], and milk urea nitrogen [MUN]) of cows in TM groups (control vs. YCW) were not significantly different (p>0.05); however, some significant variations (p<0.05) were observed in LP (early lactation vs. mid-lactation). The milk yields (kg/d, 4% FCM, ECM) of early lactating cows were significantly higher (p<0.05) than that of mid-lactating cows. Concerning milk composition and component yield, the superior milk protein content (%) (p<0.05) was observed in mid-lactating cows, while the higher yields (kg/d) (p<0.05) of milk fat, lactose, and solid non-fat were detected in early lactating cows. The SCC (10^4^/ml, log_10_) and MUN of early- and mid-lactating cows were not significantly different (p>0.05) each other.

The effects of YCW supplementation on liver enzyme activity of dairy cows in different LPs are shown in Table-2. The AST, GGT, and BUN concentrations were not significantly different (p>0.05) not only in TM group but also in LP. Before the experiment, the ALB concentration of cows in YCW group was significantly higher (p<0.05) than that of control; however, there were not different (p>0.05) after the experiment. Moreover, the ALB concentration of mid-lactating cows was also significantly higher (p<0.05) than that of early lactating cows with the supplementation of YCW.

The effect of YCW supplementation on energy metabolites of dairy cows in different LPs is presented in Table-3. Most of the energy metabolites such as BG, T-Cho, TK, ACAC, and BHB concentrations of cows were not significantly different (p>0.05) in both TM groups and LPs. Before the experiment, the early lactating cows possessed the higher value (p<0.05) of NEFA and NEFA/T-Cho ratio in compare with mid-lactating cows; however, there were not different (p>0.05) after the experiment.

The effects of YCW supplementation on blood electrolyte indices of dairy cows in different LPs are given in Table-4. The electrolyte indices such as Na, K, Cl, IP, and Mg were not significantly different (p>0.05) not only in TM groups but also in LPs. Before the experiment, the higher concentration of Ca (p>0.05) was found in early lactating cows comparison with mid-lactating cows; however, no difference (p<0.05) was observed after the experiment.

## Discussion

Early lactation in the cow is not only a period of physiologically stressful and lower immune function but also a period of high milk yield and low feed intake [1], resulted in net negative energy intake and low production performance [2]. In this experiment, milk performance in early lactating cows was greater than in mid lactating cows, consistent with a previous finding that the dietary supplementation of yeast culture and YCW increased milk production in early lactating cows [5]. This is likely due to the immunomodulatory, antioxidant, and anti-inflammatory [3] effects of YCW, which alleviated the transition stress and enhanced the immune status during early lactation. Moreover, yeast products have been shown to modify rumen function by stimulating fermentation [15] and increase populations and growth rate of cellulolytic bacteria [4], thereby improving feed utilization and enhancing animal performances such as milk production.

The average milk protein content was appeared to be lower in early lactating cows compared with mid-lactating cows. However, a similar result was obtained as the result of milk analysis before the experiment. Thus, supplementation of YCW cannot influence the milk compositions of cows, consistent with the previously published reports that milk composition appears to be unchanged under supplementation with yeast culture and YCW in early lactating cows [5]. On the other hand, the composition of milk fat, protein, and lactose was not influenced by the dietary supplementation of MOS in lactating cows [9]. The higher milk component yield occurred in early lactating cows was due to the greater milk yield of those cows after YCW supplementation.

The somatic cells are recognized as one of the major defense components of mammary glands against disease or intramammary infections. The immunomodulatory effects of YCW [3] may be beneficial to local and systemic immune responses [16], thereby decreasing SCC in milk. However, no significant effect of YCW supplementation on SCC was observed in this study, which was similar with the previous report, the therapeutic use of β-glucans infused into infected quarters had no effect on chronic subclinical mastitis [10]. It might be due to a lack of stimulation to local and systemic immunity for the reduction of SCC in dairy cows. No change of MUN observed in this study was consistent with the previous research [5], which reported that dietary supplementation of yeast products could not influence to MUN in early lactation cows.

The YCW supplementation did not affect liver enzyme activity except serum ALB; consistent with a previous finding that yeast supplementation had no significant effect on AST or GGT levels of dairy cows [17]. The higher level of serum ALB in mid-lactating cows indicates that YCW has a positive effect on liver functions of those cows. It might be due to the different energy metabolism occurred in liver of early- and mid-lactating cows. No changes of BUN observed in this experiment were supported by the previous reports [9], which stated that BUN concentration was not altered with the supplementation of yeast culture and YCW in early lactation cows.

The energy metabolites such as BG, T-Cho, TK, BHB, and ACAC of early- and mid-lactating cows were not changed by the YCW supplementation, consistent with the previous reports [8,17]. Assessing NEFA/T-Cho ratio is valuable to evaluate the energy balance and liver function of ruminants. Before the experiment, NEFA and NEFA/T-Cho ratio of cows in early lactation seems to be higher than in mid-lactation; however, these values were not different after YCW supplementation. This is likely due to a lack of body fat mobilization to supply energy demand of early lactating cows after YCW supplementation. The previous research also revealed that YCW supplementation can improve energy metabolism and nutrient utilization, thereby inhibiting lipolysis and protein catabolism [18]. Moreover, increased serum glucose levels and decreased BHB and NEFA levels observed in live yeast supplemented cows might be regarded as a sign of reduced lipomobilization processes and hepatic ketogenesis as compared with the non-supplemented control cows [19].

The blood electrolyte indices, Na, K, chloride, phosphorus, and Mg of cows in early- and mid-lactation were not affected by the supplementation of YCW, which were constant with the reports [8,17] which stated that supplementation of fermented yeast products gave no significant effects on electrolyte indices in transition cows. However, before the experiment, Ca concentration of cows in early lactation was lower than in mid-lactation, whereas no difference was observed after YCW supplementation. The researcher [20] reported that the level of Ca was elevated in dairy cows supplemented with yeast culture into the total mix ration. Thus, it could be assumed that YCW enhanced the Ca uptake of cows in early lactation rather than in mid-lactation.

## Conclusion

The positive effects of YCW supplementation on milk and milk component yields, energy metabolite, especially NEFA and NEFA/T-Cho ratio and Ca concentration were observed in early lactating cows rather than mid-lactating cows.

## Authors’ Contributions

MA and KI designed this experiment, and MA, HO, and KI carried out sample collection and analysis. MA and KI performed data analysis and interpretation. MA drafted the manuscript, and HO and KI completed the critical revision of the article. All authors read and approved the final version of the manuscript.
